# A somatotopic bidirectional hand prosthesis with transcutaneous electrical nerve stimulation based sensory feedback

**DOI:** 10.1038/s41598-017-11306-w

**Published:** 2017-09-07

**Authors:** Edoardo D’Anna, Francesco M. Petrini, Fiorenzo Artoni, Igor Popovic, Igor Simanić, Stanisa Raspopovic, Silvestro Micera

**Affiliations:** 1 Bertarelli Foundation Chair in Translational Neuroengineering, Centre for Neuroprosthetics and Institute of Bioengineering, School of Engineering, École Polytechnique Fédérale de Lausanne (EPFL), Lausanne, Switzerland; 2 The Biorobotics Institute, Scuola Superiore Sant’Anna, Pisa, Italy; 3 Specialized Hospital for rehabilitation and orthopaedic prosthetics, Belgrade, Serbia

## Abstract

According to amputees, sensory feedback is amongst the most important features lacking from commercial prostheses. Although restoration of touch by means of implantable neural interfaces has been achieved, these approaches require surgical interventions, and their long-term usability still needs to be fully investigated. Here, we developed a non-invasive alternative which maintains some of the advantages of invasive approaches, such as a somatotopic sensory restitution scheme. We used transcutaneous electrical nerve stimulation (TENS) to induce referred sensations to the phantom hand of amputees. These sensations were characterized in four amputees over two weeks. Although the induced sensation was often paresthesia, the location corresponded to parts of the innervation regions of the median and ulnar nerves, and electroencephalographic (EEG) recordings confirmed the presence of appropriate responses in relevant cortical areas. Using these sensations as feedback during bidirectional prosthesis control, the patients were able to perform several functional tasks that would not be possible otherwise, such as applying one of three levels of force on an external sensor. Performance during these tasks was high, suggesting that this approach could be a viable alternative to the more invasive solutions, offering a trade-off between the quality of the sensation, and the invasiveness of the intervention.

## Introduction

Myoelectric prosthetic hands allow upper limb amputees to regain the ability to perform several tasks involved in everyday living, representing a significant functional gain. Despite these advantages, they are often rejected by patients^1, 2^. Amongst the most common reasons cited for this reaction is the lack of sensory feedback associated with currently available prostheses, forcing users to rely on vision to guide their movements^3^. This problem is so pronounced, that some users prefer to use body powered prostheses, where the cables used to actuate the limb provide some rudimentary form of indirect sensory feedback^4^.

One of the major goals in the development of future upper limb prostheses is thus the restoration of sensory feedback. Several benefits have been associated with the addition of touch, and include the improved ability for users to integrate the external limb as their own^5, 6^ and the possibility to perform certain tasks that might otherwise be arduous (e.g., precise control of prosthesis force)^7^.

Sensory feedback strategies can commonly be classified along two dimensions. First, approaches can be categorized as either non-homologous, where feedback is provided via a different sensory modality (e.g., electro-tactile or vibro-tactile stimulation of the skin to convey pressure), or homologous, where the restored sensation matches the original sensation (e.g., invasive electrical stimulation of the sensory fibers innervating the hand)^8^. Secondly, approaches can be categorized as either somatotopic, where the induced sensations are felt as originating from the correct region (e.g., sensation is felt on the phantom limb), or non-somatotopic, where the sensations are felt in an unrelated region (e.g., vibro-tactile stimulation of the skin on the arm to convey touch events on the hand)^9^.

The ideal solution is to restore sensory information using a homologous and somatotopic approach because of the inherent simplicity and intuitiveness, which allows for immediate and effortless incorporation of the feedback within the sensory-motor scheme. This is in stark contrast with non-somatotopic approaches, which necessarily introduce the need for training.

Several recent studies have demonstrated the effectiveness of homologous and somatotopic approaches in human patients using implanted neural interfaces^7, 10–12^. These patients could distinguish the shape and stiffness of objects and perform precise motor tasks. Although the functional results obtained in these studies are promising, several significant obstacles still need to be overcome before such solutions can gain widespread clinical adoption, such as chronic implant stability and system miniaturization^13^. Even if these solutions become widely available, they will still require an invasive intervention, which might not be indicated for certain amputees, or which the patient might simply not wish to undergo. For these reasons, there is considerable interest in developing a novel approach that might combine the advantages of a somatotopic sensory restitution scheme, while at the same time mitigating some of the drawbacks usually associated with implantable approaches, such as the need for surgery.

Non-invasive, somatotopic sensory feedback approaches have been demonstrated in the past using mechanical or electrical stimulation of the stump^14, 15^. However, in order to achieve a somatotopic scheme, these studies have relied on the presence of a hand map on the stump (where touching certain areas of the stump induces referred sensations to the phantom limb). Unfortunately, such hand maps are not present in all amputees (two independent studies found that roughly 65% of trans-radial amputees have some form of hand map), and when present, may not always be complete, with only some fingers represented^16, 17^. To the best of our knowledge, no study currently exists in the literature proposing a non-invasive, somatotopic feedback system and testing it in amputees with a closed-loop prosthesis.

In addition to the somatotopic approaches mentioned above, several studies have extensively tested and discussed non-invasive, non-somatotopic approaches using mechanical^8, 18, 19^, electrical^8, 20, 21^ or even auditory^22, 23^ feedback modalities. Such non-somatotopic strategies have been demonstrated to lead to higher difficulty in interpreting sensations, denoted by an increase in response time, lower discrimination accuracy, and longer learning periods^9, 24^. There are therefore several reasons to develop a non-invasive, somatotopic feedback approach which does not rely on the presence of a hand map on the stump, and could thus benefit the entire amputee population.

To address this lack, we developed a non-invasive and somatotopic sensory feedback approach based on TENS. We first characterized the sensations elicited by TENS in four trans-radial amputees^25, 26^. We then implemented a non-invasive, bi-directional prosthesis based on TENS, in which electrodes placed on the skin could activate underlying nerves and generate conscious sensations of paresthesia referred to the phantom limb. Using this setup, we asked the subjects to perform several functional tasks to evaluate the potential increase in prosthesis use performance offered by the addition of sensory information in the human-machine interface.

## Results

### Elicited sensation characterization

We first performed an in-depth characterization of the elicited sensation in all four subjects by exploring the stimulation parameter space and recording the intensity, location and quality of the sensations.

A sensation of paresthesia referred to the phantom limb was elicited in all four subjects. All subjects described the sensation as an unnatural feeling over the hand, mentioning sensations such as tingling or vibration (one patient described the sensation using the following words: “it feels like when you have a speaker over the skin and the bass vibrates”). However, the subjects were also quick to integrate this information as a touch-like sensation, often making comments such as “I was *touched* here” while pointing at the experimenter’s hand. This indicated in a qualitative way that although the sensation did not resemble natural touch, the subjects quickly and intuitively interpreted the sensation they perceived as a form of touch.

**Figure 1:**
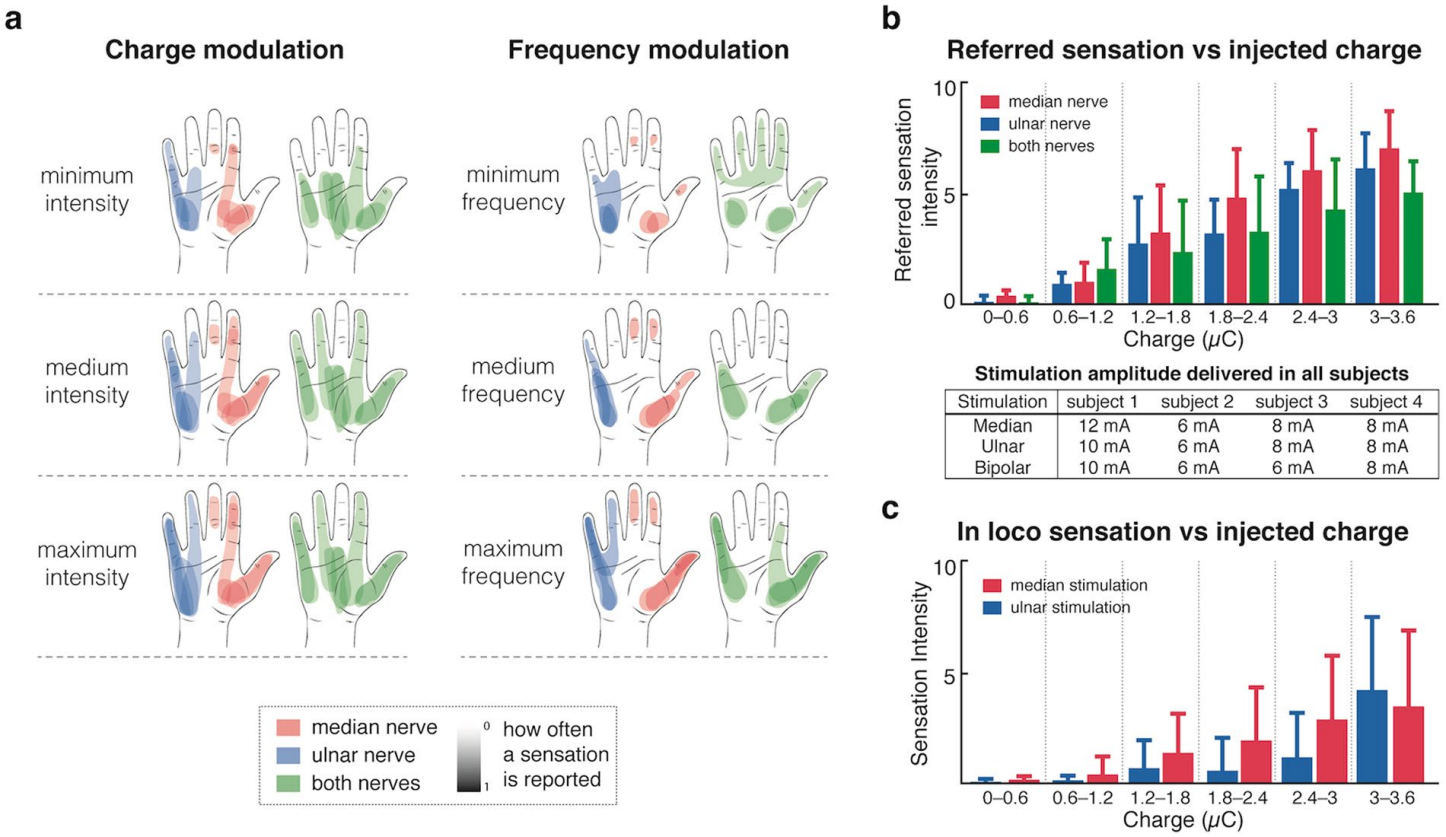
Characterization results for four subjects. (**a**) shows the areas of elicited sensation as described by the patients. The three rows show the variation of the reported area as the applied charge or frequency are modulated. The intensity of the coloring represents how often an area was reported (dark coloring means all patients felt a sensation in the corresponding location, while a lighter coloring indicates a region only felt by a smaller proportion of subjects). The reported areas were extracted from the drawings done by the subjects using the software interface. (**b**) shows the reported referred sensation intensity (scale from 1–10) according to the stimulation pulse width (amount of injected charge). A table reports the stimulation currents used in all subjects for the different stimulation channels. (**c**) shows the evolution of the in loco sensation (under the electrode) as current is increased. Both bar plots are represented with standard deviation (n = 4 subjects).

Figure 1 reports the results of the characterization test. Two topographically distinct sensations were elicited, depending on where the electrodes were placed (either over the ulnar nerve, or over the median nerve). The distinction was clear in all patients: stimulating the ulnar nerve always resulted in a distinctly different sensation to that obtained when stimulating the median nerve, and both corresponded to the natural hand innervation areas of the respective nerves.

TENS also always resulted in a local sensation under the stimulating electrode in addition to the distally referred sensation (in-loco sensation). Depending on the location of the stimulating electrodes, the intensity of the in-loco sensation compared to the referred sensation changed. With optimal electrode positioning, it was possible to obtain a weak “in-loco” sensation, which did not distract the subject from the referred sensation. However, it was never possible to remove the in loco sensation completely. The optimal positioning was very subject dependent, and was usually attained empirically by moving the electrodes around the starting position until the reported sensation resulted in a clearly defined referred sensation to the phantom hand, which was easy to distinguish from the “in-loco” sensation.

The region of elicited sensation and the intensity of the sensation changed when modulating the amount of injected charge or the stimulation frequency. When varying the amount of injected charge (modulating the pulse width), the subjects reported a proportional increase in perceived sensation intensity (Fig. 1b) and small changes to the area of the sensation (Fig. 1a). Surprisingly, when varying the stimulation frequency, the subjects reported a change in the area of elicited sensation (Fig. 1a), as well as in the intensity of the sensation. Because of the variability in the area of elicited sensation using frequency modulation, pulse width modulation was used to deliver tactile information during bidirectional prosthesis use. Sensation quality did not change significantly as we modified the various stimulation parameters, and was always perceived as paresthesia.

**Figure 2:**
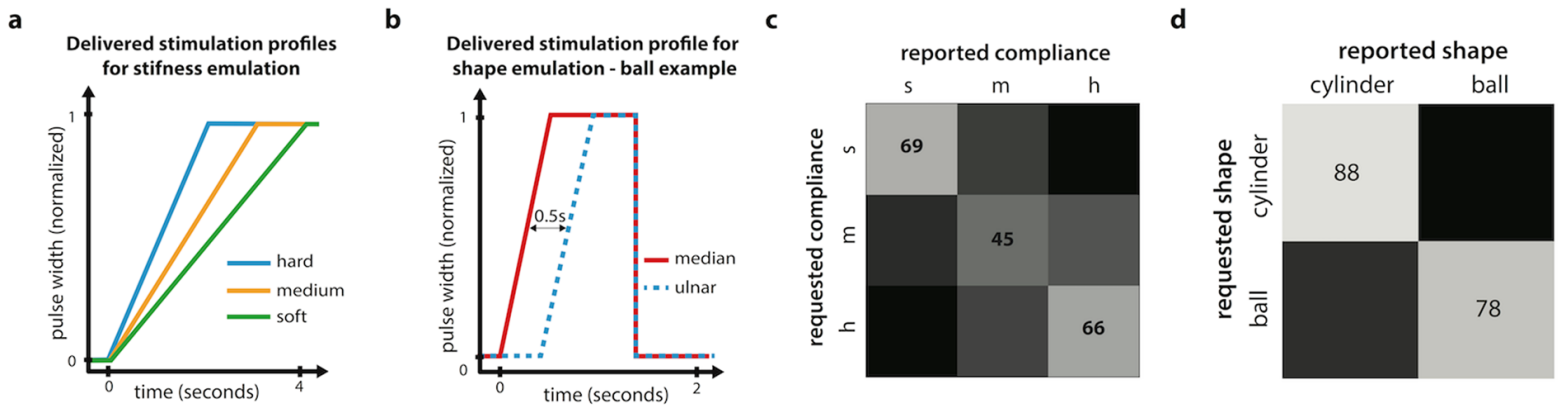
Compliance and shape recognition. (**a**) and (**b**) show the delivered stimulation profile used to emulate stiffness and shape respectively. These profiles were extracted from a previous study and were matched to the experimental force profiles measured in that work. (**c**) shows the confusion matrix for the identification of the three compliances profiles delivered. (**d**) shows the confusion matrix for the identification of the two shape profiles. For all the virtual objects tasks, three subjects performed the experiments. The results shown are the average from all subjects. For compliance recognition subjects 1, 2 and 3 performed respectively 93, 59 and 59 repetitions while for shape recognition 39, 34 and 94. h = hard, m = medium, s = soft.

To study the ability to perceive temporal changes in stimulation parameters, we delivered time-dependent patterns of stimulation designed to replicate the sensations felt during manipulation of various objects (with varying compliance or shape). When such dynamic stimulation profiles were delivered, Subjects 1, 2 and 3 could recognize the different virtual objects above chance level. Figure 2 shows the stimulation profiles, as well as the overall performance for the two types of tasks. In the first task, which consisted in determining if a virtual object was “hard”, “medium” or “soft”, the overall task performance was 60% correct answers (chance level for this task was 33% correct answers). The subjects reported difficulties in performing this task, stating that the difference between the objects was not always very clear. Although the subjects did many errors, they very rarely confused a hard virtual object for a soft one, or the opposite (Fig. 2c). Most errors were done between adjacent levels (e.g., soft confused with medium, and medium with hard).

The second task, which consisted in differentiating between a spherical virtual object (delay between the contact of the different finger segments) and a cylinder (simultaneous contact on all fingers), was reported as much easier to perform. This was reflected in a higher performance for this task (83% of correct answers, whereas chance level for the task was 50% correct answers).

### EEG recordings

**Figure 3:**
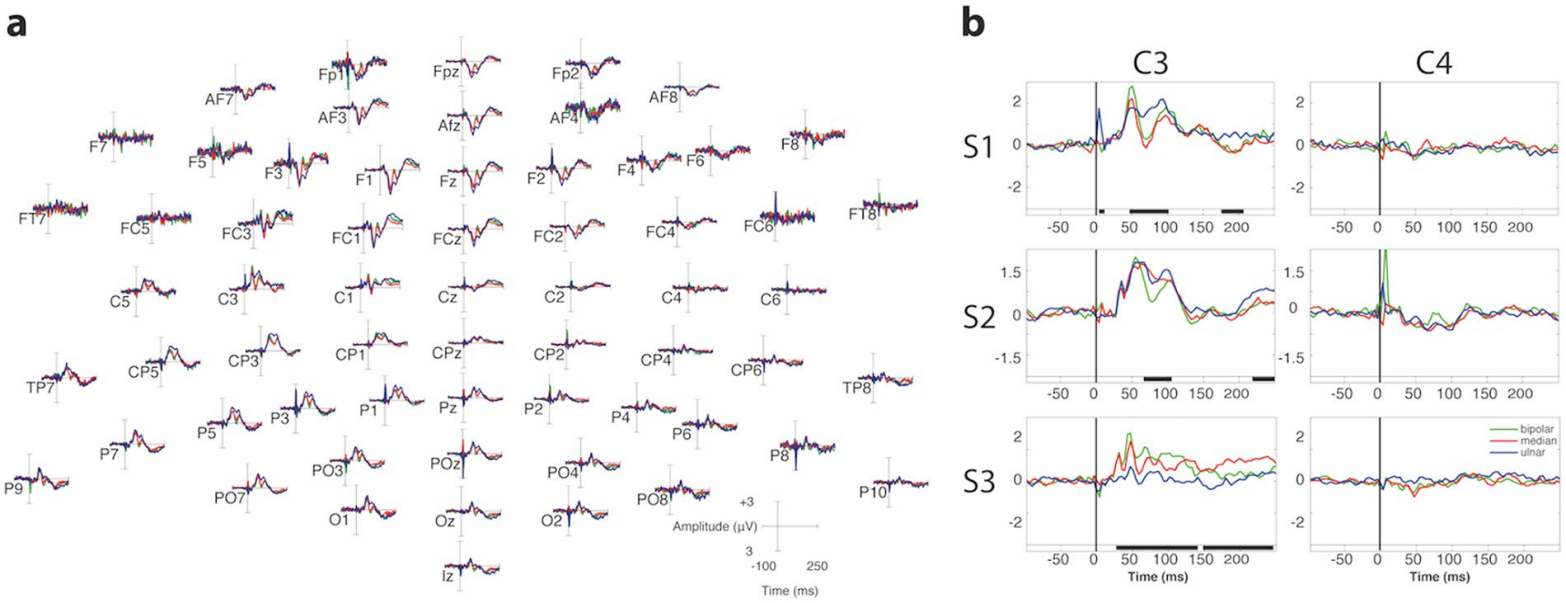
EEG data for all subjects. (**a**) shows the grand-average event related potentials (ERPs) for bipolar (blue), median (red) and ulnar (green) electrical stimulation in the scalp topography of the first subject. ERPs range from −100 ms to 250 ms time locked to the onset of the stimulation. (**b**) shows the grand-average event related potentials (ERPs) for bipolar (blue), median (red) and ulnar (green) electrical stimulation in contralateral (channel C3) and ipsilateral (channel C4) average-referenced derivations with statistics. Significant differences (as revealed by cluster statistics, see methods) are marked in black at the bottom of each panel. The timeline is referenced to the onset of the stimulus. S1 = subject 1, S2 = subject 2, S3 = subject 3.

EEG data was acquired concurrently with TENS stimulation of either channels (ulnar and median) or both together. Figure 3a shows the grand-average event related potentials (ERPs) for the first subject. The electrical stimulation elicits clear somatosensory evoked potentials (SEPs), mainly distributed contra-laterally to the stimulated hand. Figure 3b shows a comparison of SEPs elicited by bipolar, ulnar and median nerve stimulation in contralateral (C3) and ipsilateral (C4) derivations. SEPs as early as 35 ms (Subject 3) or 45–50 ms (Subjects 1 and 3) are significantly modulated by the type of stimulation, in contralateral derivations only. For subjects 1 and 2, a separate interval around 200 ms with respect to the stimulus onset is also modulated. Subject 3 is characterized by significant contralateral SEP differences throughout the whole 35 ms–200 ms interval.

**Figure 4:**
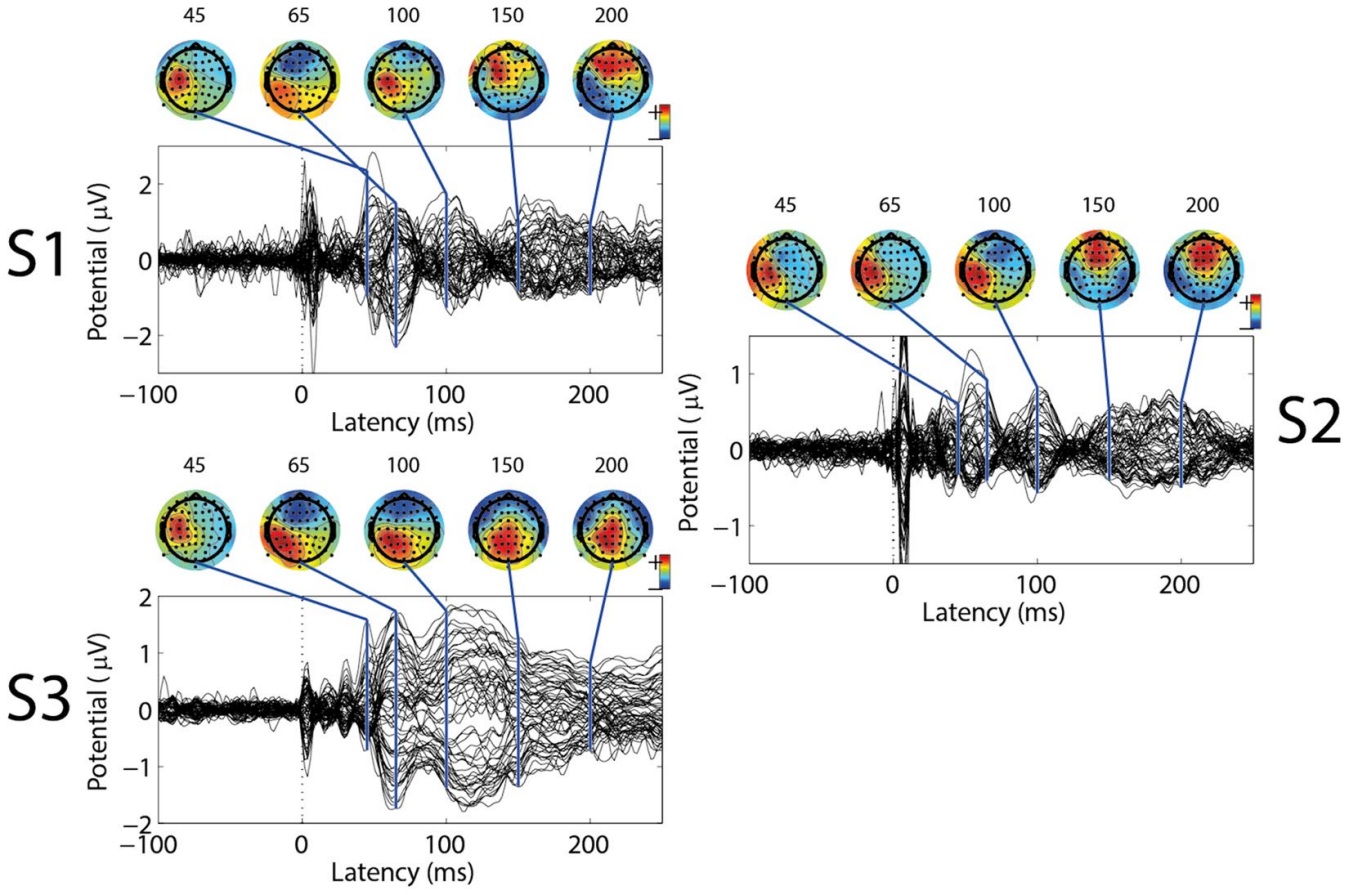
EEG data by subject. Butterfly plots of the grand-average event related potentials (ERPs) for subjects 1, 2, and 3 during bipolar stimulation. Topographies are represented at latencies of 45, 65, 100, 150 and 200 ms with respect to the stimulus onset.

In addition, scalp topographies (Fig. 4) are consistent with a generator localized at the post-central gyrus. Additional cortical regions, such as posterior parietal and frontal cortices are activated after 100 ms with respect to the stimulus onset, and are marked by a posterior parietal P100 and bilateral frontal N140 in Fig. 4.

### Functional tasks

To study the performance of the proposed TENS-based bidirectional prosthesis setup, we performed three functional tasks involving both motor control and sensing, putting the subjects in situation reminiscent of everyday living tasks.

**Figure 5:**
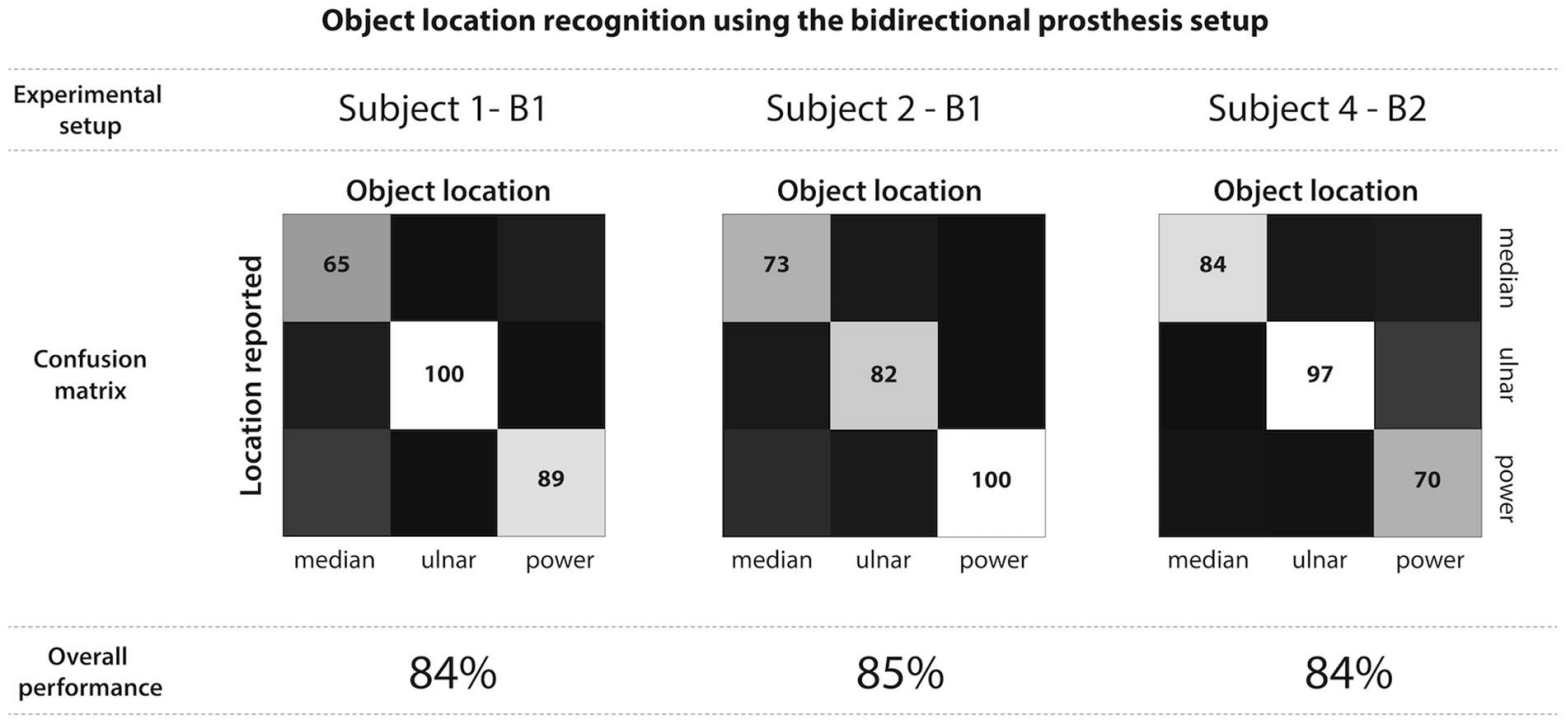
Confusion matrices for object location task. Detailed confusion matrices are shown for the object location task for three subjects. The first row indicates which experimental setup was used. The second row shows the performance over all trials, shown as a confusion matrix. The third row indicates the overall task performance for each subject. The total number of repetitions was n1 = 50, n2 = 33 and n4 = 30, for subjects 1, 2 and 4 respectively.

In the location recognition task, subjects were asked to close their hands around an object, and determine if it had been placed in the ulnar side of the hand, the median side or across the entire hand. All three subjects tested could perform this task with high accuracy (84–85% correct answers, as opposed to a 33% chance level), as reported in Fig. 5. This high level of performance indicated that the sensations elicited through stimulation of the ulnar or the median nerves were easily distinguishable for all subjects. Additionally, these results confirmed that there is very little crosstalk between the two stimulation channels, as previously reported in the characterization tests.

In the force generation task, subjects were asked to grasp a dynamometer with either a low, medium or strong grip force. We first tested four healthy control subjects, who were all able to successfully generate three different levels of force with high accuracy (average 93% correct levels). When asked to generate four distinct levels of force, the subjects often complained that the task was hard and that they were unsure about how well they were performing. Although performance was lower (75% correct levels on average) they were still able to perform this task successfully (capable of generating four statistically different levels of force) except for Subject 2. Indeed, Subject 2 had a much harder time controlling the output force, and obtained poor performance compared to the other subjects, both when generating three and four levels of force (73% and 57% respectively). Subject 1 also asked to try generating 5 distinct levels of force in a separate trial, and was successful in generating 5 statistically different levels of force with relatively high performance (72% correct levels).

Three subjects performed the force generation task. Two different experimental setups were tested, which we will call B1 and B2 in the rest of this manuscript. The difference between the B1 and B2 setups was the way we handle stimulation artifacts in the sEMG signals. B1 was based on software multiplexing, while B2 was based on hardware blanking (additional details on these two approaches and the motivation for both are given in the materials and methods).

**Figure 6:**
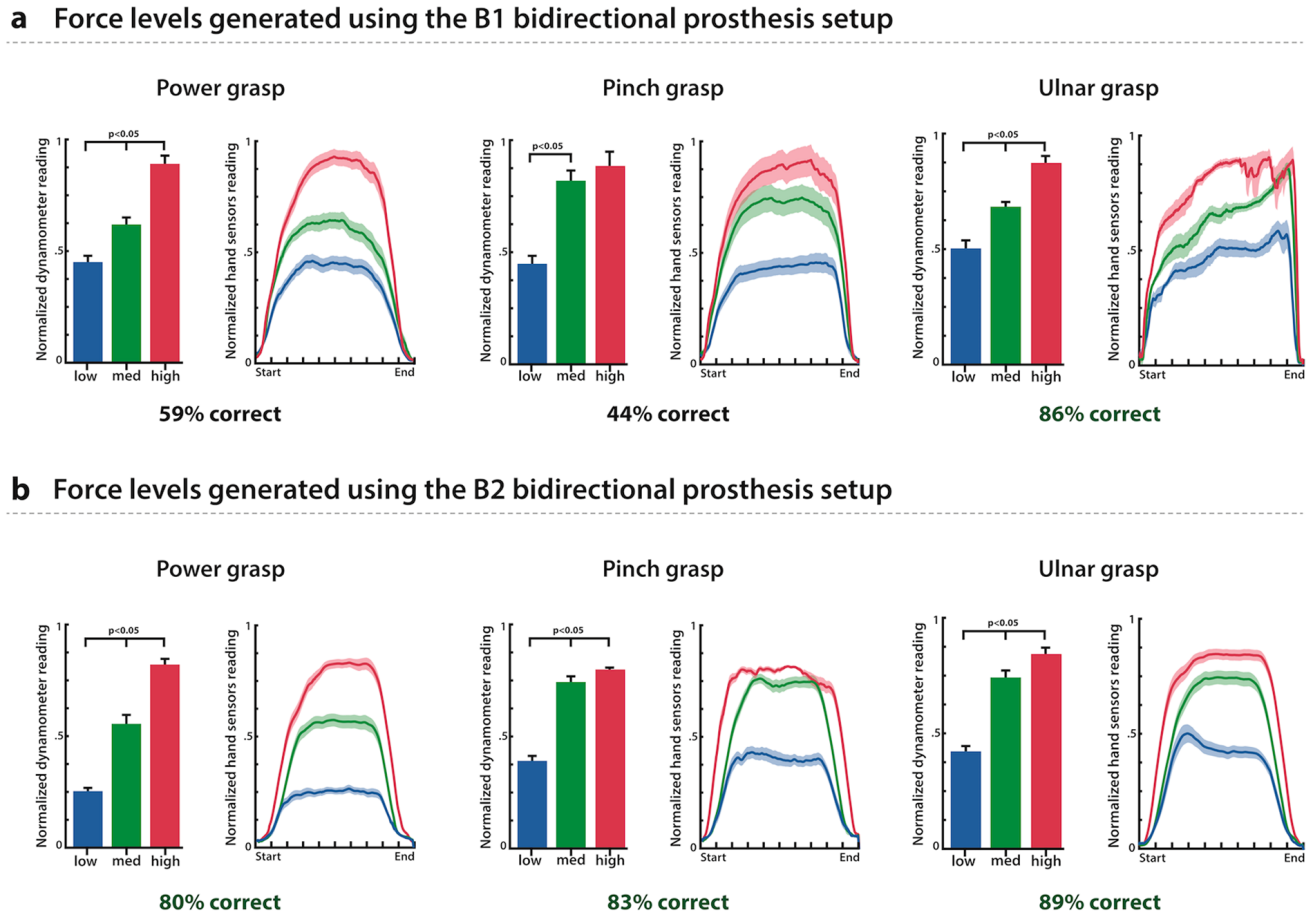
Overall results for the force levels generation task. **(a**) shows the force profiles measured from the hand’s internal force sensors, as well as the average force reached for each of the three force levels performed when using the B1 prosthesis setup for all grasping types. Also reported are the overall performance levels. (**b**) shows the same results as (**a**) but for the B2 prosthesis setup. The B1 setup was tested with 2 subjects, while the B2 setup was tested with one subject. For the B1 setup, n = 186 repetitions were done with the power grasp, n = 106 with the pinch grasp and n = 33 for the ulnar grasp (all subjects included). For the B2 setup, n = 120 repetitions were done with each grasp type. The bar charts are reported with standard error, and the shaded areas in the plots represent +/− standard error.

All three subjects could generate three statistically different levels of force using three types of grasping patterns (ulnar, median and power grasps), as reported in Fig. 6, with the only exception being the median grasp (also referred to as pinch grasp) when using the B1 setup. Using B1, the overall performance obtained for all grasping types was: 59% of correct trials for power grasp, 44% for pinch grasp and 86% for ulnar grasp. The subject using the B2 setup was able to achieve higher levels of performance, with an average percentage of correct trials of 84% (80% for power grasp, 83% for pinch grasp and 89% for ulnar grasp).

**Figure 7:**
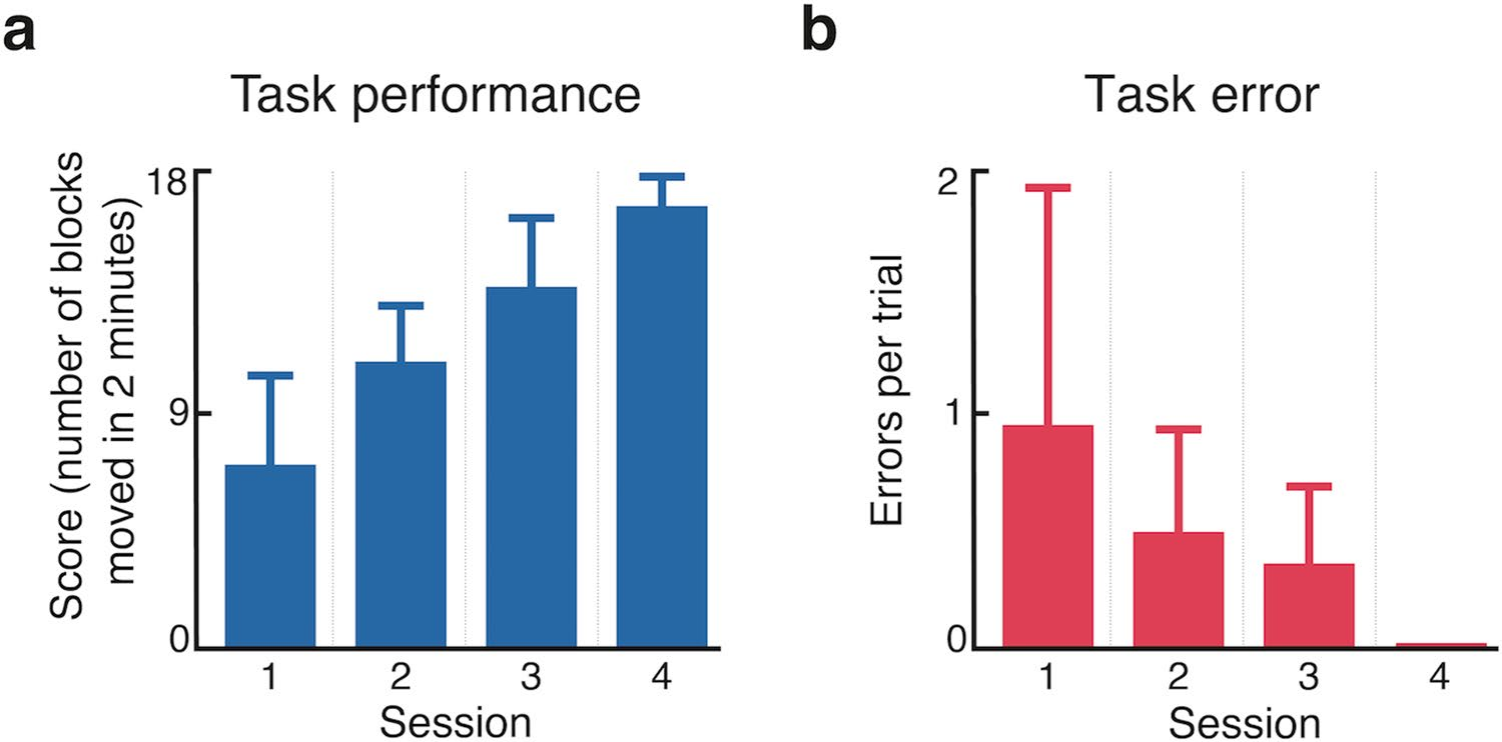
Performance during “sensory blocks” test. (**a**) shows the average score (points per 2-minute trial) for two subjects for each session. (**b**) shows the average number of errors for two subjects across sessions (errors per 2-minute trial). All bar charts are reported with standard deviation. A total of n = 24 trials were performed (6 two minute trials per session).

We then investigated the performance of bidirectional prosthesis setup, in a more complex functional task with two subjects (Subjects 1 and 2). In this task (“sensory blocks”), subjects were asked to move as many blocks as possible over a central separation during a 2-minute period. The blocks were not always placed in the hand, thus forcing the subjects to rely on their artificial sense of touch to decide whether or not to move their hand to the other side. Figure 7 shows the results obtained during this task. The two subjects quickly increased their overall performance as they performed more and more sessions. Indeed, the average score in the first session was 8 (corresponding to eight blocks moved within a period of two minutes), while after four sessions, the mean score moved up to 17 in the same period of time. Similarly, the mean number of errors decreased from session to session as the subjects gained confidence with the setup. In the last session, no errors were observed.

## Discussion

### Pulse width modulation with TENS is a suitable candidate for prosthesis sensory feedback

This study showed that TENS can be used within a bidirectional prosthesis setup to restore a referred sensation on the phantom limb, and to improve performance during motor tasks. We extensively characterized the changes in reported sensation as we explored the stimulation parameter space. We found that although the area of elicited sensation may not easily be modified in a controllable way, the strength of the induced sensation can be very well controlled (Fig. 1a). This makes TENS a suitable candidate for restoring graded information of touch to prosthesis users.

Overall, within single channels, there was only a very limited possibility to modulate the area of sensation when modulating the intensity of the stimulation. This seems to indicate that the delivered stimulation quickly activates most of the large diameter fibers whose receptive fields cover large areas of the phantom hand. Still, the activation of smaller diameter fibers, whose receptive fields were likely superimposed with the previously recruited afferents, was enough to achieve sensation intensity modulation. Our results are thus in accordance with findings from Kuhn *et al*. who showed it is possible to modulate motor fiber recruitment using superficial stimulation^27^.

The reported areas of sensation rarely corresponded to the whole region of innervation for the stimulated nerve (Fig. 1a). Although it appears that almost all the hand is covered, some areas, such as the middle finger, show a very light coloring, indicating that only one patient reported a sensation on that finger). The likely mechanism underlying these observations is the lower current needed to stimulate superficial fibers within the nerve (closer to the stimulating electrode), as opposed to deeper structures^28^. Our subjects would thus be expected to report muscle twitches or painful sensations in the areas innervated by superficial fibers, before reporting sensory perceptions in areas innervated by fibers deeper within the nerve, or further from the electrode.

Additionally, cortical remapping occurring after amputation^29^ may have extensively altered the brain’s response to touch afferents coming from the phantom limb. Stimulating peripheral sensory fibers may no longer elicit the appropriate response within the sensory cortex.

Finally, our results do not appear to be in accordance with the results reported recently by Chai *et al*., who were able to elicit selective referred sensations of touch on single fingers. We argue that the dissimilarities in these two works arise from a subtle difference, which might not be apparent at first. Although both works are based on TENS, the two techniques are targeting different underlying structures. In our work, we are using TENS to stimulate the underlying nerve as a whole, in an approach similar to what has been done previously with more invasive techniques^7, 10^. Instead, Chai *et al*. are targeting local cutaneous afferents in the underlying skin. Since the skin, in their case, may have been naturally re-innervated by sensory fibers, they are able to elicit referred sensations of selective touch (which can also be achieved by simply touching the subjects on the stump in these cases). Although more selective, their approach requires that such peripheral reorganization has taken place, which holds true only in a portion of the amputee population^15^. In our case, we did not find any such sensibility in any of the four subjects included in our study.

### Frequency modulation with TENS modulates the area of the reported sensation

An unexpected result was the observation that the area of the reported sensation changed when modulating the stimulation frequency. Since the firing rate of sensory afferents encodes sensation intensity^30^, and not sensation area (this is encoded at the population level, where each afferent reports touch events in its receptive field), we would not expect frequency modulation to have any effect on the reported area. One hypothesis is that the capacitive components of the epidermis and the electrode-gel junction, which confer a frequency dependent property to the system’s impedance, may cause variations in the voltage field within the tissue with varying stimulation frequency, resulting in modified fiber recruitment patterns. A thorough modelling study may be required to better explain these results.

### Precise, open-loop sensory tasks are possible using TENS

To complete our characterization, we performed two simulated tasks in open-loop (with no prosthesis control). In these two tasks, we emulated the stimulation profiles that would be generated when touching objects with different properties. We reported that all tested subjects could sense the emulated compliance or shape of a virtual object. Our results suggest that our system would potentially be able to provide basic compliance and shape information to the users.

The EEG results, despite limitations in terms of spatial resolution, suggest that the tactile sensation generated by the three types of provided stimulation elicit early and late SEP components. Early SEP components around 40–70 ms were characterized by a positive deflection on parietal contralateral channels (e.g. C3) and a negative deflection on frontal channels (e.g. FC1) symmetrically distributed across the CPz–C1–FC3 line (Fig. 3a). Scalp topographies (Fig. 4) of early stage potentials confirm the compatibility of early stage SEPs with a generator localized at the post-central gyrus (Broadmann areas 2 and 3) and are consistent with a physiological tactile activation of the primary and secondary somatosensory cortices^31–33^. Late Somatosensory Evoked Potentials (SEPs) are compatible with bilateral generators in the frontal lobes, including orbito-frontal, lateral and mesial cortex^34^, which confirms the secondary somatosensory cortex (SII) support of bilateral tactile representation also seen in^33, 35^. The compatibility of elicited SEPs with literature and the significant differences among stimulation modalities seem to confirm the prevalence of a main referred sensation. This by no means excludes contamination by non-somatotopic in-loco sensations, but suggests a representation of touch in the brain compatible with the referred sensation reported by the subjects. The topographical differences across subjects in late SEPs (140–300 ms) can be attributed to the physiological differences in high level processing of the stimuli among subjects.

Zhang *et al*. adopted EEG to quantify vibration and pressure sensation with different intensities elicited by electrical cutaneous stimulation at varying frequencies of the middle finger, in healthy subjects^36^. They demonstrated that only late components (i.e. 200–300 ms) and topographical differences successfully encode the stimulation modality (vibration vs pressure). In our work, we used a different stimulation paradigm (single pulse), a different region of superficial stimulation (stump) and a different population (amputees). However, we showed similar topographies and confirmed that SEPs amplitudes can discriminate different stimulation modalities (median, ulnar, bipolar) as soon as 50 ms after stimulus onset, even in amputees.

### Subjects can localize where an object touched the hand

We reported that all three subjects who used the bidirectional system could successfully distinguish touch events happening on either the ulnar side of the hand, the median side or both at the same time. Performance at this task was high across all subjects, indicating that the restored sensation is perceived clearly enough to accurately judge whether the touch occurred in the median region of the hand, the ulnar region or both at the same time. In other terms, this stimulation method allows for some basic spatial selectivity by selecting which channel to inject charge from.

Furthermore, the performance in the location discrimination task remained unchanged regardless of the different experimental setups used (B1 or B2). This result is expected, since this simple task does not depend on the timing of the delivery of the sensation. Indeed, the user simply closed the robotic hand, and waited for a sensation. If the sensation was delayed (as was the case when using the B1 setup), the subject simply had to wait longer until he could determine where the object was located, but no additional errors were introduced.

### Subjects can generate three statistically different levels of force

When using their prosthesis with sensory feedback turned on, all subjects could generate three statistically different levels of force consistently (with one exception being the pinch grasp when using the B1 setup). These results indicate that the sensory information provided by means of TENS was sufficient to reliably understand the amount of force being applied to an external object within a bidirectional setup. It is important to note that although the open loop characterization had indicated that TENS was able to provide graded sensation over a range of values (patient used a 10-point scale to rate the intensity), this did not necessarily imply that the sensation would remain as clear during an active control task (we speculated that interfering muscle activity and reduced attention to the sensation may affect performance).

Additionally, we measured the performance of healthy subjects at the same task, to establish a baseline success rate to compare our results against. Healthy subjects were easily able to generate three different levels of force very reliably (of all four subjects tested, three could perform this task with 100% accuracy, while one had lower performance). When asking for four different force levels, the performance dropped significantly, indicating that even for healthy subjects, this task is not trivial. We argue that the limiting factor when increasing the number of requested force levels is cognitive (e.g. remembering exactly what force was used previously for level 2 as opposed to level 4), rather than related to motor control or sensory feedback. These results indicate that although prosthesis users can reach high levels of performance at this task when using sensory feedback (73% correct overall for three force levels), there is still progress to be made before reaching the same level of performance achieved by healthy individuals (93% overall for the same task).

### Increase in control and stimulation delays may degrade performance

The difference between the two setups (B1 and B2) became apparent in the force levels generation experiment. Our results for these tests indicate that the B2 setup resulted in overall higher task performance. The additional delay introduced in the B1 setup resulted in lower performance, confirming that increases in system delay may negatively impact prosthesis control performance^37^. Special care must be taken in insuring low overall system delay when implementing bi-directional prosthetic solutions, to minimize the negative effects introduced by the use of higher delays. No statistical analysis was performed to support these observations regarding the difference between the two setups, because only one subject used the B2 setup. Further experiments would be needed to confirm these preliminary observations.

Although hardware blanking (B2) resulted in a lower maximum stimulation frequency, in our case this did not impose a practical difference since the stimulation frequencies used were relatively low. However, this may be an important consideration when choosing between the two approaches if higher stimulation frequencies are required. A further practical consideration is that hardware blanking (B2) may not be widely available, while software multiplexing (B1) is a more universal approach which could be implemented using any existing system.

### Subjects displayed unexpected behavior when performing complex motor sensory tasks

During the “sensory blocks” task, on some trials the subjects would drop the object as they were moving it from one side to the other. Although they had received no instruction regarding this specific scenario, the reactions we observed were surprising and indicative of the users relying heavily and intuitively on their artificial sense of touch. For instance, one subject announced, immediately after dropping the object, that it had slipped and that he wished to begin a new trial, moving his hand back to the starting position. In a similar scenario, sometimes the subjects did not let go of the object (insufficient open command) and started moving back to the starting position. In these cases, the subjects would quickly realize their mistake and bring back the object to the correct side, and release it.

Such examples of unexpected interactions between the amputee and their environment constitute further evidence supporting the idea that an intuitive sensory restoration scheme (somatotopic) may much more easily be understood and incorporated by the subject, leading to a faster learning phase and a better outcome.

### A feasibility study for a new type of non-invasive somatotopic bidirectional prosthesis

The experimental system described in this work constitutes, to the best of our knowledge, a first experimental proof of a non-invasive, somatotopic bi-directional prosthesis in upper-limb amputees. Several systems for non-invasive feedback have been proposed in the past, using such techniques as vibro-tactile and electro-tactile stimulation of the stump or other body regions^38, 39^. However, the novelty of the approach proposed in this work resides in the use of non-invasive stimulation techniques (here TENS) to elicit somatotopic sensations referred to the phantom limb, without relying on the presence of a phantom hand map on the stump. We argue that delivering somatotopic sensations is preferable, since it has been shown to lead to a more intuitive system requiring a shorter learning phase and shorter response times^9, 24^. This may in turn translate into increased prosthetic limb control performance. Additionally, prosthesis embodiment has been shown to increase when the sensory inputs coming from different modalities are congruent^5^. In this case, having a referred sensation of touch that is spatially congruent to the site of physical contact (as seen visually by the subject) may lead to an increase in prosthesis embodiment, which has several beneficial effects for the user. However, objective measurements regarding robotic hand embodiment would need to be performed to confirm the hypothesis that in this scenario, somatotopic feedback increases prosthesis embodiment.

Demonstrating the real-world feasibility of this approach provides stronger clinical evidence of the potential benefits compared to simply demonstrating the availability of the individual components in isolation. This is particularly true in the case of upper limb prosthetics, where often single components are not tested on amputees, relying on indirect results obtained in healthy subjects instead^40^, or by performing experiments in virtual environments presented on screen^38^. Although the individual components used in this study have been described to some extent in the literature (even if not extensively in the case of TENS for eliciting referred sensation to the phantom limb), demonstrating the clinical feasibility of a combination approach constitutes a novel and important step forward. Some of the effects described in this work could only be observed by combining sensory feedback and sEMG based control within a closed-loop setup. Indeed, many of the results reported here arise from an interesting interplay between control strategies and sensory feedback.

### Comparison of TENS based feedback to invasive sensory feedback strategies

It may be of interest to compare our results to previous studies where invasive stimulation electrodes (TIMEs and cuff electrodes) were used to elicit touch in a bi-directional prosthesis setup^7, 10, 11^. Indeed, what was demonstrated in this study may be proposed as a suitable alternative to more invasive approaches, for instance for patients who may not qualify for surgery, for patient who may prefer not to undergo surgery, or for scenarios where lower costs may be required (low income countries). Since no standardized tests exist to quantify the closed-loop performance of a prosthetic limb (or are not widely used), we may only compare our results qualitatively.

Using our system, subjects could determine where a touch event had occurred on the robotic limb (they could recognize one amongst three possible regions of touch). This ability was similar to what had been reported in the literature previously^7^. Furthermore, the subjects from this study could control the output force of the robotic hand to generate three statistically different levels of force. This was directly comparable to the results obtained by Raspopovic *et al*., since we did not find significantly lower performance in this task between the two studies, while in Tan *et al*. such a quantitative measure is missing. Finally, regarding the experiments we performed in open-loop, where we asked our subjects to identify the stiffness and shape of virtual objects, the reported performance using TENS was lower than what was found using neural electrodes in previous studies. Although both techniques allowed users to perform this task successfully, invasive neural stimulation resulted in higher task performance.

Long-term stability remains an open question for invasive technologies. TENS on the other hand, offers a solution which is likely to remain functional for as long as necessary, if stimulating electrodes are replaced when needed, like sEMG electrodes. Electrode placement is critical in obtaining suitable referred sensations. However, once the positions were found, we had no difficulty re-placing the electrodes before every session, and this step would be further improved if the electrodes were directly integrated within the socket. Once the electrodes are placed on the skin and under the socket, they are unlikely to become sufficiently displaced to disrupt proper functioning of the system. In our experiments, we never observed a loss of referred sensation during arm movements in space (such as during the “sensory blocks” task).

There are certain aspects where the non-invasive alternative falls short of its invasive counterpart. The biggest concern in this regard is the quality of the sensation. Even though invasive interfaces do not always elicit natural sensations of touch (subjects often report paresthesia, or other slightly more natural sensations, such as vibrations or pressure waves), the reported sensations are much closer to a naturally elicited touch than what we report with TENS. For example, Tan *et al*. reported a “natural pressure perception” when using certain stimulation paradigms, and Raspopovic *et al*. reported sensations as corresponding to the “physiological sensory mapping of touch”^7, 10^. We were able to test TENS on one subject who had previously undergone invasive nerve stimulation (Subject 4). Although this is an isolated observation, it may be of interest. This subject reported that the sensation elicited through TENS was in no way comparable to the sensation he had previously experienced with an implanted electrode. He described the sensation obtained using TENS as “much less natural”. Furthermore, the stimulation artefacts induced by TENS and measured in the sEMG signals represent an additional technical challenge which is not encountered when using invasive bidirectional prostheses.

The possibilities offered by TENS are much more limited in terms of selectivity, modulation, and naturalness of the induced sensation compared to what can be achieved using invasive technologies. These limitations may potentially be improved with novel approaches, such as by using an array of stimulating electrodes combined with a beamforming approach to improve selectivity, or using more sophisticated encoding approaches to obtain natural sensations or to offer a wider range of modulation. However, the limitations of TENS are likely to remain challenging. Additionally, it is unlikely that TENS could provide the type of fine sensation described by Oddo *et al*. using implanted intra-neural electrodes^31^. Nevertheless, the actual functional performance of our system, in the scenarios tested, was comparable to the performance reported in previous studies using invasive approaches, indicating that subjects using our non-invasive bidirectional prosthesis did not have severe functional disadvantage compared to users using invasive alternatives.

It remains undisputed that invasive approaches may provide much more function in the future. Current studies on invasive methods for restoring sensory information have not exploited the richness of the restored sensation to its full potential. Only future studies may truly reveal the extent to which neural interfaces will be able to restore natural and complete sensations to the users.

## Materials and Methods

### Patient recruitment

Four subjects participated in the study (36 years old male with a distal right arm amputation, 29 years old male with a distal right arm amputation, at the level of the wrist junction, 38 years old female with a distal right arm amputation, 36 years old male with a distal left arm amputation). All four patients had very distal amputations, situated close to the wrist.

Ethical approval was obtained by the cantonal Ethical committee of Vaud, and the Specialized Hospital for Rehabilitation and Orthopedic Prosthetics in Belgrade, and informed consent was signed by all volunteers. During the entire length of our study, all experiments were conducted in accordance with relevant guidelines and regulations. In addition, specific informed consent was obtained for publication of identifying images when relevant.

### Elicited sensation characterization

TENS was applied over residual median and ulnar nerves, to elicit a referred sensation on the phantom hand. TENS was delivered using an electrical stimulator commonly used for functional electrical stimulation (RehaStim, Hasomed, Germany). The stimulator delivered square charge balanced biphasic pulse trains, with controllable amplitude (steps of 2 mA), pulse width (steps of 20 μs) and frequency (externally imposed).

**Figure 8:**
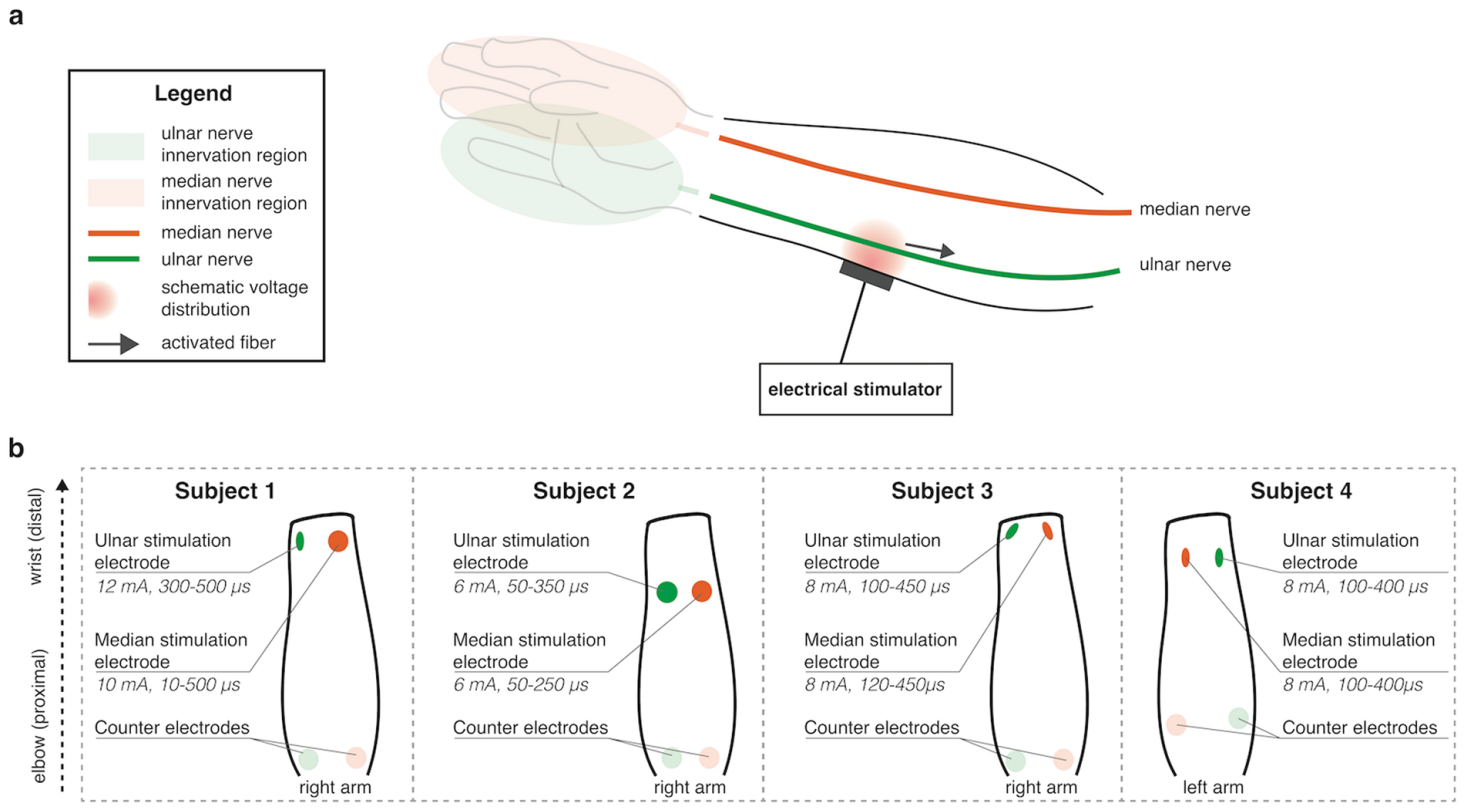
Transcutaneous electrical nerve stimulation (TENS) setup. (**a**) schematic representation of transcutaneous electrical nerve stimulation in the context of upper limb sensory restitution. This figure schematically illustrates how an electrode placed on the skin can generate a voltage field within the residual forearm’s soft tissue. By placing the electrodes in the appropriate positions, the voltage field can elicit referred and clear sensations from the missing hand, corresponding to median and ulnar nerve innervations, as shown in transparent green and red. This allows for a certain amount of selectivity in the elicited response. (**b**) shows the exact electrode placements for each of the four subjects. The stimulation parameters (fixed amplitude, range of pulse widths) are also shown. The positions of the electrodes were patient specific.

By placing electrodes on the skin (PALS neurostimulation electrodes, Axelgaard, US) in specific areas where the underlying nervous structures are close to the surface of the skin and easily accessible, it is possible to elicit activation of hand afferents, leading mainly to a paresthesia reported over the phantom limb (Fig. 8a). Initially the stimulating and return electrodes were round with a radius of 2.5 cm. In some cases (Subjects 1, 3 and 4), the round stimulation electrodes were cut with scissors to a more oval shape, which resulted in a smaller contact surface with the skin.

Figure 8b shows the precise electrode positioning used for each of the four subjects. The electrode placement and stimulation parameters were calibrated during an initial extensive exploration phase.

To fully characterize the quality, intensity, location and extent of the elicited sensations, a characterization test (mapping or calibration procedure) was performed, using a custom designed graphical user interface (LabView). During this procedure, an extensive parameter search in the stimulation variables space was performed, varying one of three parameters (pulse width, pulse intensity or pulse train frequency). More specifically, we first found the range of acceptable stimulation currents for each subject using a fixed pulse width of 250 µs. Since the stimulator did not allow for very fine control of the stimulation intensity (steps of 2 mA), this step was usually very short, and resulted in two or three acceptable values for the intensity. Then, for each stimulation current found in the first step, the pulse width was varied from 0 to 500 µs with steps of 20 µs. For every set of parameters, each subject was asked to describe the quality of the elicited sensation using one of the proposed keywords (tingling, vibration, natural touch, pulsing) or their own words. Additionally, the subjects were asked to rate both the sensation directly under the electrode (in-loco), and the referred sensation (on the phantom hand) using a visual-analog scale ranging from 1 to 10. Finally, the subjects were required to draw the region corresponding to the induced sensation on a picture of a hand and forearm. When either the local or referred sensations were reported as too strong or produced muscle twitches, the stimulation was stopped.

A second test (emulation of compliance) was performed to explore how well the subjects could understand the time course of the provided sensation. In this experiment, three different electric current profiles were delivered in open loop. These three profiles (ramps) were trains of pulses where the amplitude increased with different speeds. The durations of the ramps were extracted from a previous study^7^, and corresponded to three objects with different compliances (the faster the increase, the lower the compliance) (Fig. 2a). These three profiles were extracted from a previous study from Raspopovic *et al*., where they were obtained using a prosthesis and three every-day object with different compliances^7^. These three profiles represent a subset of the possible interactions between a prosthesis and object encountered in activities of daily living. The patients were instructed to imagine that they were pressing on an object, and to announce whether the object was hard, medium, or soft.

A third experiment (emulation of shape) was performed using a virtual ball and a virtual cylinder, where the cylinder induced simultaneous activation of the ulnar and median channels, while the ball activated the ulnar channel with some delay compared to the median, replicating the way a ball might first come in contact with some fingers, and only later with the rest of the hand as the fingers close around the object (Fig. 2b). Subjects 1, 2 and 3 performed all three experiments, while Subject 4 only performed the first one.

During both open-loop experiments described above, the subjects were allowed a short familiarization session (approx. 1–2 minutes) during which they received each type of stimulation together with an explanation of what it represented (e.g. “this is a hard virtual object”). Once this short session was over, the recorded experiments started, during which each answer was recorded to compute task performance. A minimum of 30 repetitions per subject were performed for each type of experiment.

### Electroencephalographic recordings

Neural correlates of transcutaneous electrical nerve stimulations were investigated by acquiring 64-channel electroencephalographic (EEG) data. Three amputees were recruited and underwent one rest session (10 minutes) and three stimulation sessions (median nerve, ulnar nerve and both nerves simultaneously) lasting 20 minutes each. During stimulation, the inter-stimulus interval was set to 700 ms, allowing an average of 1500 stimuli per session. Subjects were instructed to fix a point in front of them, to keep their facial muscles relaxed and to avoid sudden head movements.

Signals were recorded using a 64 channel EEG device (ActiveTwo, Biosemi B.V., Amsterdam) with a 2 kHz sampling rate. The montage was in accordance to the 5% 10/20 system^41^. Electrode impedance was kept below 10 kΩ in at least 95% of derivations throughout the experiment. Data were analyzed with Matlab scripts based on the EEGLAB toolbox^42^. To maximize dipolarity and the reliability of the extracted Event Related Potentials (ERPs), EEG signals were processed using the Reliable Independent Component Analysis (RELICA) method to remove non-neural sources of noise and other artefacts before epoching^43, 44^. To optimize the ICA decomposition the raw data were high-pass filtered using a zero-phase, 1 Hz, 24^th^ order, Chebyshev type II filter to increase stationarity and low-pass filtered using a zero-phase, 45 Hz, 70^th^ order, Chebyshev type II filter before being resampled at 256 Hz. Channels with probability more than five times the standard deviation or with prominent artefacts (as confirmed by visual inspection)^45, 46^ and remaining channels were average-referenced. More than 55 channels remained for every subject. Epochs containing high-amplitude artefacts or high-frequency muscle noise were removed. The remaining data was submitted to RELICA with Infomax^47^ as core and 100 point-by-point bootstrap repetitions. The ICA decomposition was saved and re-applied to the raw data, which was then high-passed using a 0.5 Hz, 94^th^ order, Chebyshev type II filter and a custom 50 Hz comb notch filter^48^. RELICA allowed reliable identification of stereotyped artefacts such as eye movements and eye blinks, which were removed from the data. Epochs ranging from −100 ms to 250 ms, and time locked to the onset of each stimulation pulse were extracted. Noisy epochs were rejected by careful visual inspection. As with continuous data, the criteria for epoch removal was the presence of high amplitude artefacts (e.g., Jaw clenching). Trials were normalized using the pre-stimulus baseline average^49^. ERP’s statistical significance between stimulation types (ulnar, median, bipolar) was assessed using a Montecarlo statistic with cluster correction for multiple comparisons^50^, adapted from the FieldTrip toolbox^51^. Scalp topographies were drawn directly from ERPs by associating to each channel location its channel value at a defined latency, color coded by amplitude (blue – negative values; red – positive values; green – null values).

### Bidirectional setup

**Figure 9:**
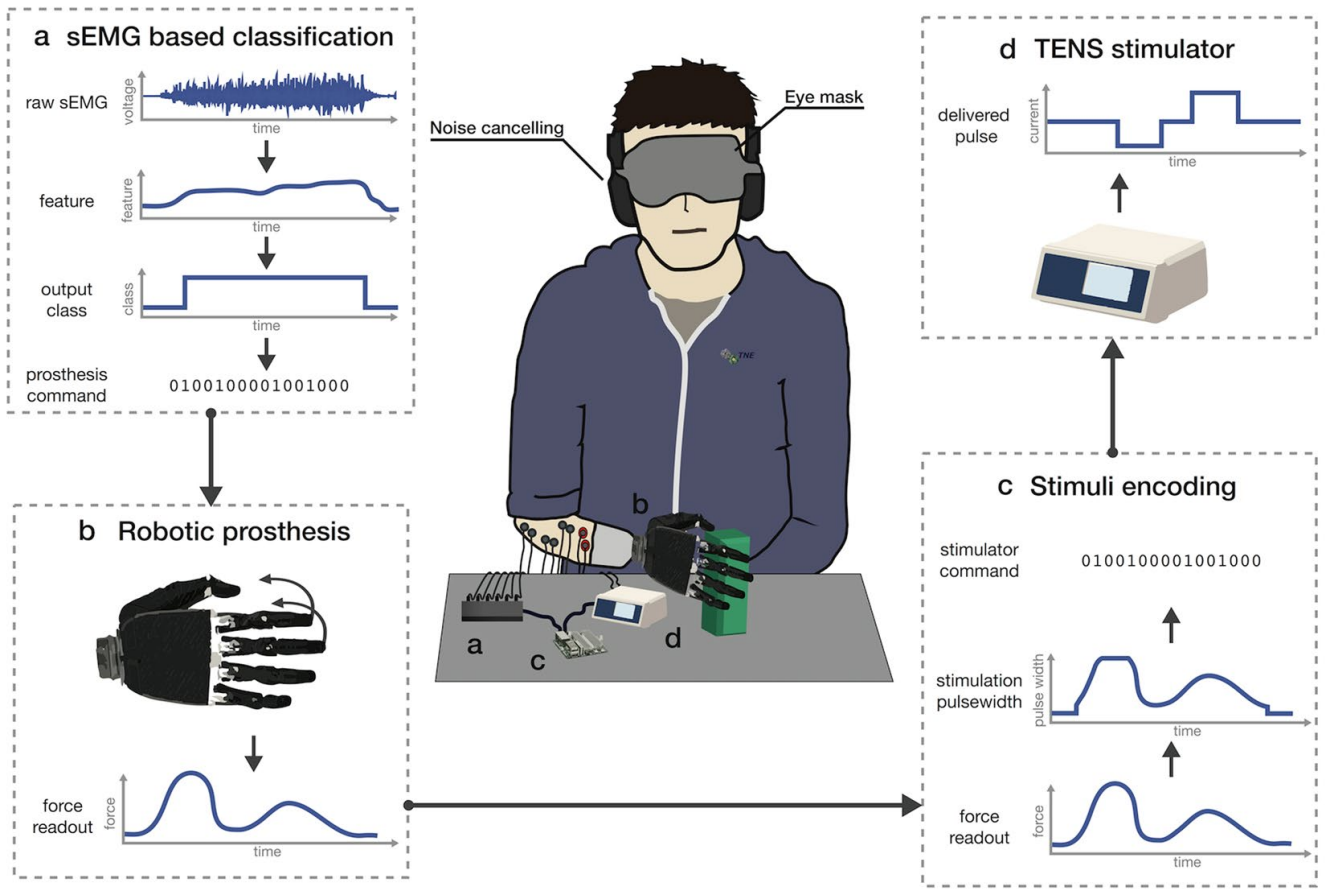
Schematic overview of the bidirectional experimental setup components. First (**a**) the sEMG signal is acquired from the residual stump muscles. Features are extracted from the signals and the classifier predicts the desired output class. The corresponding command is sent to the robotic hand (**b**), which moves accordingly. A force signal is measured from the robotic fingers sensors. This signal is then processed by the embedded computer (**c**), which computes the appropriate stimulation signal to elicit the desired sensation. This signal is then sent to the stimulator (**d**), which delivers the stimulation pulses to the subject’s stump via stimulation electrodes.

An overview of the bidirectional prosthesis setup can be seen in Fig. 9. To implement a fully bi-directional hand prosthesis setup, we integrated the sensory feedback modality within a myoelectric control scheme. The resulting setup allowed the subject to control a robotic hand using his/her residual muscle activity, while at the same time receiving relevant tactile information by means of TENS. To achieve this integration, the bidirectional prosthesis setup was based on a combination of custom designed hardware and software, as well as commercially available components. The Hasomed surface stimulator was connected to a central single board computer (Odroid U3, Hardkernel) where a custom, multithreaded C++ code was running. Also connected to this central device were a multichannel surface electromyography (sEMG) recording apparatus (Neural Interface Processor, Ripple, US) and a robotic hand with integrated tension sensors fitted to each finger (Prensilia Azzura, Prensilia, Italy). Stimulation intensity (pulse width modulation) was associated to the output from the corresponding hand sensors in such a way as to optimally cover the whole dynamic range of sensations reported by the subject. Since sensors were present on each finger, but only two areas of stimulation were used (median and ulnar), the maximum value from the sensors for each area was used (first three fingers for median, last two for ulnar). The relationship between hand sensor readout and stimulation pulse width was as follows: when the sensor reached a minimum value (set just above the sensor noise floor), the pulse width was set to its minimal value (corresponding to a very light, but perceptible, sensation). Then, pulse width varied linearly with the hand sensor readout until it reached its maximum value (corresponding to the strongest sensation below pain).

For the control, a standard pattern recognition based controller with 5 classes was used^52^. In our case, we used the following classes: median grasp (closing the three first fingers of the hand, also referred to as pinch grasp), ulnar grasp (closing the last two fingers of the hand), power grasp (closing all fingers), open (opening all fingers) and rest (no movement at all). The classification was based on a 4 channel EMG recording from the patient’s residual muscles (palpation was used to identify muscle groups which were active during specific grasping patterns). sEMG data were acquired with a sampling frequency of 1 kHz. The signal was filtered using an IIR filter with 4^th^ order Butterworth characteristics, between 15 and 375 Hz. An additional notch filter was used to remove 50 Hz noise. Our classification windows were set to 100 ms, which has been shown to be a good value for classification accuracy^37^. The robotic hand was controlled using position control with the following approach: every 33 ms, the robotic hand’s finger positions would increment in the direction of the last decoded grasp type (e.g. if power grasp was the last decoded class, all fingers would close a little more (each “step” is approximately 1.5°), if open was the last, all fingers would open a little more). This approach resulted in a smooth movement of the hand. In the case of contact with an object, prosthesis output force thus increased progressively over time as a given class continued to be decoded. We extracted a single feature per channel (i.e. mean absolute value). A training dataset for the classifier was acquired every time the electrodes were applied, in which the subjects were asked to hold each class successively for three seconds. They were instructed to use a level of contraction which was comfortable for them. The classifier (K-nearest neighbors classifier, with K = 3) was then trained and the same parameters were used until the electrodes were removed or until performance degraded noticeably.

Particular attention was devoted to address issues due to the appearance of large stimulation artefacts in the EMG signal. Since stimulation electrodes and EMG recording electrodes are placed in proximity on the same forearm, the severity of these artefacts can be very high. This problem has been studied extensively in the context of electro-cutaneous feedback, and several software and hardware approaches have been proposed to mitigate some of the complications these artefacts may occasion^40, 53^.

In the first experimental setup (B1), software time division multiplexing was used^53^. This approach consists in dividing time into two types of windows: stimulation windows, in which stimulation is delivered and no EMG data is recorded, and classification windows, in which no stimulation is delivered and EMG data is recorded and classified. The transient response caused by the stimulation artefact reaching the amplification stage, lasted up to several hundreds of milliseconds. Correspondingly, long durations for each stimulation window (300 ms) were chosen to allow all stimulation artefacts to settle before switching to a recording window. The overall delay in resituating sensory information after a touch event was, in the worst case, 0.4 seconds when using the B1 setup.

In the second experimental setup (B2), hardware blanking was used as a method to remove stimulation artefacts^40^. In this approach, EMG data was continuously acquired, and small segments of signal were removed every time a stimulation pulse was delivered. In our case, 20 ms of signal were removed starting right before each stimulation pulse. When using a stimulation frequency of 30 Hz, the maximum feedback delay obtained using the B2 setup was approximately 35 ms, considerably lower than with the B1 setup. Such a low value of delay is not perceptible to the user^37^. However, the drawback of this approach is that maximum stimulation frequency is limited.

During bidirectional prosthesis experiments, stimulation parameters were set as follows: stimulation frequency was fixed at 50 Hz for B1, and 30 Hz for B2. In both cases, amplitude and pulse-width were calibrated independently for each patient based on the characterization results.

Subjects 1 and 2 performed the functional experiments using the B1 setup, while Subject 4 performed the experiments using the B2 setup. Subject 3 was unable to perform any functional experiments, because she was unable to generate reliable control commands due to strongly atrophied muscles.

### Wearable system and custom sockets

For some of the functional experiments requiring the subject to move the robotic limb independently, custom molded sockets were built with an integrated screw to easily fix the robotic hand on the end. Holes were drilled in the appropriate positions to allow for the placement of sEMG and stimulation electrodes on the stump. The hardware for the setup was entirely battery powered, and could either be worn by the subject in a small backpack or be placed on a table nearby.

### Functional tasks

In the first functional experiment (object location recognition), subjects were asked to close the robotic hand using their voluntary muscle activity. When the hand was closed, an object was presented to the hand in either of three positions: over the whole hand, in the ulnar region (little finger and ring finger) or in the median region (thumb, index finger and middle finger). After each trial, the patients had to announce where the object had been placed (full hand, median or ulnar position). The subjects were blindfolded and acoustically isolated to ensure that they were not relying on any external cue to make their judgment. Since this task was very intuitive, the subjects did not perform a familiarization session for this experiment. Instead, we directly initiated the recorded trials. A minimum of 30 repetitions per subject were done.

In the second functional experiment (generation of different force levels), the robotic hand was placed against an external dynamometer (hand dynamometer, Vernier, US). Any force the hand generated was measured by the sensor. Subjects were instructed to close the hand and generate one of three levels of force (low, medium or high). They were instructed to rely on the sensory information to judge how much force they were applying and to stop when they considered they had reached the desired level. The subjects performed a short familiarization session (approx. 5 minutes), during which they could squeeze the dynamometer with their prosthesis and the experimenter made sure they had understood the task. A minimum of 30 repetitions per subject were performed for each type of experiment.

To obtain a comparison point, for this experiment, we asked four healthy subjects to perform a force generation task using their dominant healthy hand. To explore the abilities of healthy subjects, we performed the experiment twice, asking for either three or four levels of force. Each subject performed a trial consisting of 10 repetitions per force level, asked in a random order.

In a third experiment (“sensory blocks” test), subjects were asked to perform a more complex functional task in which the hand was connected using a custom-built socket. In this task, patients were sitting in front of a table divided in the middle by a 15 cm high plane. They were asked to close their robotic hand whenever they wished. If they felt an object, they were instructed to grab the object securely, and move it from the left side of the table to the right side, making sure to pass above the separation. They then dropped the object on the other side, and came back to the starting position. If no object was felt, the subjects were instructed to reopen the hand and start over. Objects were placed in the hand 80% of the time. Each trial lasted for two minutes, during which a point was attributed for every block that was successfully moved. Additionally, a point was also awarded when the subjects correctly identified that the object slipped from the hand and took actions to correct it. Errors were recorded when the subject moved the hand despite no object being presented, or when the object slipped and the subject took no corrective action (did not notice it). This task allowed us to observe the evolving and emerging behavior that the subjects displayed when confronted with an everyday life task. This task was separated into four different sessions, each with a minimum of 3 repetitions per subject (each repetition lasting 2 minutes). There was no familiarization session, and performance was evaluated independently for each session to search for learning effects.

### Data analysis and statistics

The data from all experiments were extracted in Matlab (R2014b, The Mathworks, Natick, US), where all the analyses were performed. Statistical tests were applied when appropriate. The results for the statistical tests, as well as the relevant metrics (number of repetitions, type of test performed, significance) are reported alongside the corresponding figures. Unless otherwise stated, a statistical level of significance of 0.05 was used. For the analysis of the force levels (Fig. 6), a one-way ANOVA test with a Tukey-Kramer post-hoc test for multi group comparison was performed. For each trial, the duration was normalized, and an average force value was computed over a fixed interval (interval from 60% to 90% of trial completion). To compute the “performance” score (given as a percentage of correct trials), we first obtained the average force value for each force level, using the method outlined above. Then, we assigned each repetition to the nearest force level. Finally, we computed the performance score as the percentage of repetitions correctly assigned to the right force level. In order to ascertain that the data was normally distributed and allowed the use of the ANOVA, a one-sample Kolmogorov-Smirnov test was performed.
